# Heteronemin Induces Anti-Proliferation in Cholangiocarcinoma Cells via Inhibiting TGF-β Pathway

**DOI:** 10.3390/md16120489

**Published:** 2018-12-06

**Authors:** Hung-Yun Lin, Shu-Leei Tey, Yih Ho, Yung-Tang Chin, Kuan Wang, Jacqueline Whang-Peng, Ya-Jung Shih, Yi-Ru Chen, Yung-Ning Yang, Yu-Cheng Chen, Yi-Chang Liu, Heng-Yuan Tang, Yu-Chen SH Yang

**Affiliations:** 1 Graduate Institute of Cancer Molecular Biology and Drug Discovery, College of Medical Science and Technology, Taipei Medical University, Taipei 11031, Taiwan; linhy@tmu.edu.tw (H.-Y.L.); jqwpeng@gmail.com (J.W.-P.); 2 Taipei Cancer Center, Taipei Medical University, Taipei 11031, Taiwan; yutangchin@gmail.com (Y.-T.C.); Wangk@tmu.edu.tw (K.W.); yajungshih@yahoo.com.tw (Y.-J.S.); aquarlus9132@yahoo.com.tw (Y.-R.C.); 3 TMU Research Center of Cancer Translational Medicine, Taipei Medical University, Taipei 11031, Taiwan; 4 Traditional Herbal Medicine Research Center of Taipei Medical University Hospital, Taipei Medical University, Taipei 11031, Taiwan; 5 Pharmaceutical Research Institute, Albany College of Pharmacy and Health Sciences, Albany, NY 12208, USA; tang506@gmail.com; 6 Department of Pediatrics, E-DA Hospital, Kaohsiung 824, Taiwan; djsr2000@hotmail.com (S.-L.T.); ancaly@yahoo.com.tw (Y.-N.Y.); 7 School of Medicine, I-Shou University, Kaohsiung 824, Taiwan; 8 School of Pharmacy, Taipei Medical University, Taipei 11031, Taiwan; yiho@tmu.edu.tw; 9 Cancer Center, Wan Fang Hospital, Taipei Medical University, Taipei 11031, Taiwan; 10 Graduate Institute of Nanomedicine and Medical Engineering, College of Medical Engineering, Taipei Medical University, Taipei 11031, Taiwan; 11 The Ph.D. Program for Cancer Biology and Drug Discovery, China Medical University and Academia Sinica, Taichung 404, Taiwan; j520c1@gmail.com; 12 Division of Hematology-Oncology, Department of Internal Medicine, Kaohsiung Medical University Hospital, Kaohsiung 807, Taiwan; ycliu@cc.kmu.edu.tw; 13 Department of Internal Medicine, Faculty of Medicine, College of Medicine, Kaohsiung Medical University, Kaohsiung 807, Taiwan; 14 Joint Biobank, Office of Human Research, Taipei Medical University, Taipei 11031, Taiwan

**Keywords:** heteronemin, cholangiocarcinoma, TGF-β pathway

## Abstract

A marine sesterterpenoid-type natural product, heteronemin, retains anticancer effects. In the current study, we investigate the antitumor mechanism of heteronemin in cholangiocarcinoma cells and further explore its molecular targets. Initially, heteronemin exhibited potent cytotoxic effects against cholangiocarcinoma HuccT1 and SSP-25 cells. In vitro, heteronemin altered the abilities of cell adhesion and cell migration in HuccT1 and SSP-25 cell lines. It repressed messenger ribonucleic acid (mRNA) expression levels of transforming growth factor (TGF)-β, mothers against decapentaplegic homolog (SMAD) and Myc, whose protein products play important roles in regulating cell growth, angiogenesis, and metastasis. In addition, heteronemin altered several signaling pathways. The results indicate that heteronemin was able to modulate cell adhesion, the expression of extracellular matrix (ECM) receptors, the TGF-β pathway, cell motility, the membrane integration, metastasis response, matrix metalloproteinase (MMP) remodeling, the regulation of metabolism, sprouting angiogenesis, transcription factors, and vasculogenesis in cholangiocarcinoma cell lines. The results also suggest that it activated multiple signal transduction pathways to induce an anti-proliferation effect and anti-metastasis in cholangiocarcinoma. In conclusion, heteronemin may be used as a potential medicine for anticancer therapy.

## 1. Introduction

Cholangiocarcinomas are malignant tumors of the biliary tract and are the second most common type of primary liver cancer [1]. An increasing incidence of cholangiocarcinoma has been documented. It is epidemiologically important throughout the world, but effective chemotherapy for this tumor is not yet available [1,2]. Cholangiocarcinomas have universally poor outcomes, with surgical resection offering the only choice for treatment. Furthermore, cholangiocarcinomas are associated with a high mortality rate because they are difficult to detect early. In addition, cholangiocarcinoma is resistant to most chemotherapeutic agents. Cisplatin or gemcitabine has been used as a standard chemotherapeutic agent for cholangiocarcinoma. However, recent studies have found that several gemcitabine-resistant cell lines are cross-resistant to 5-fluorouracil (5-FU), doxorubicin, and paclitaxel, indicating their multidrug-resistant nature [3]. On the other hand, gefitinib has been reported to be a radiosensitizer, which inhibits the radiation-induced phosphorylation of epidermal growth factor receptor (EGFR) and the downstream pathway, and therefore enhances radiosensitivity in cholangiocarcinoma cells [1,2].

Recently, our studies indicated that lovastatin inhibits the proliferation of cholangiocarcinoma cells via inhibition of the expression of transforming growth factor (TGF)-β1, cyclooxygenase (COX)-2, and intercellular adhesion molecule (ICAM)-1 [4]. In addition, lovastatin down-regulates the expressions of integrin β1 and integrin β3. Acting independently, integrin β3 and liver kinase B1 (LKB1) play important roles in the inhibition of proliferation by lovastatin in human intrahepatic cholangiocarcinoma. These results suggest that (TGF)-β1 may be involved in the regulation of proliferation in cholangiocarcinoma.

TGF-β is a pleiotropic cytokine that plays different roles in cancer progression. It can act as a tumor suppressor, regulating the transcription of tumor suppressor genes, inhibiting cell proliferation, and promoting cell cytostasis, apoptosis, and autophagy in early stages of cancer progression [5,6,7,8]. TGF-β-induced senescence in vivo is associated with a strong antitumor response against hepatocellular carcinoma [9]. By activating a mammalian target of rapamycin via the phosphoinositide 3-kinase/AKT pathway, TGF-β promotes the translation of ubiquitin carboxyl-terminal hydrolase 15 (USP15). Via deubiquitination, USP15 binds to stabilize p53 [6]. TGF-β signaling starts upon the binding of TGF-β to its receptor, TGF-βRII, after which it forms a heterotetrameric complex with TGF-βR1. Subsequently, it further phosphorylates TGF-βR1 by TGF-βRII. The activated TGF-β receptors recruit and phosphorylate transcription factors SMAD2 and SMAD3 [10,11,12]. The phosphorylated SMAD2/3 form a complex with co-SMAD (SMAD4) to translocate into the nucleus as co-activators to form a transcription complex for regulating the expression of numerous target genes [13,14,15].

Heteronemin, the most plentiful secondary metabolite in the sponge *Hippospongia* sp., shows effective cytotoxic activity against different cancer cells. It increases the percentage of apoptotic cells and reactive oxygen species (ROS) in Molt4 cells [16]. Furthermore, heteronemin-induced production of ROS from the mitochondria and apoptosis can be suppressed [16] by the ROS scavenger, N-acetyl cysteine (NAC) [17]. Heteronemin has been shown to increase talin expression and the accumulation of phosphorylated talin in Molt4 cells, but it is only able to increase phosphorylated talin in human embryonic kidney 293 (HEK293) cells [16]. Treatment of the ROS scavenger reverses heteronemin-induced talin activation. Conversely, restricted evidence is available concerning the results of talin phosphorylation in cancer cells. Heteronemin has been shown to be a farnesyl transferase inhibitor (FTI) which suppresses the cytarabine-induced, farnesyl transferase-mediated activation of Ras [18], as well as the activation of downstream signal transduction pathways such as mitogen-activated protein kinases (MAPK), activator protein 1 (AP-1), nuclear factor-κB (NF-κB), and c-Myc. Heteronemin interferes with actin microfilament and causes morphology changes [16]. Heteronemin is able to induce cytotoxic effects via oxidative stress and the induction of phosphorylated talin expression [16].

In this study, we investigated the anti-proliferative effect of heteronemin and mechanisms involved in human cholangiocarcinoma cell cultures. We found that heteronemin induced anti-proliferation in human cholangiocarcinomas. Heteronemin inhibited the expression levels of TGF-β, SMAD, and Myc messenger ribonucleic acid (mRNA). Heteronemin was also able to modulate several signal transduction pathways and regulate cell adhesion, the expression of ECM receptors, the TGF-β pathway, cell motility, the integral membrane, metastasis response, MMP remodeling, the regulation of metabolism, sprouting angiogenesis, transcription factor, and vasculogenesis in cholangiocarcinoma cell lines. In summary, heteronemin might be used as a potential medicine, either alone or in combination with other anticancer drugs, to treat cholangiocarcinomas.

## 2. Results

### 2.1. Heteronemin Inhibited Cell Proliferation of Cholangiocarcinoma Cells In Vitro

**Figure 1:**
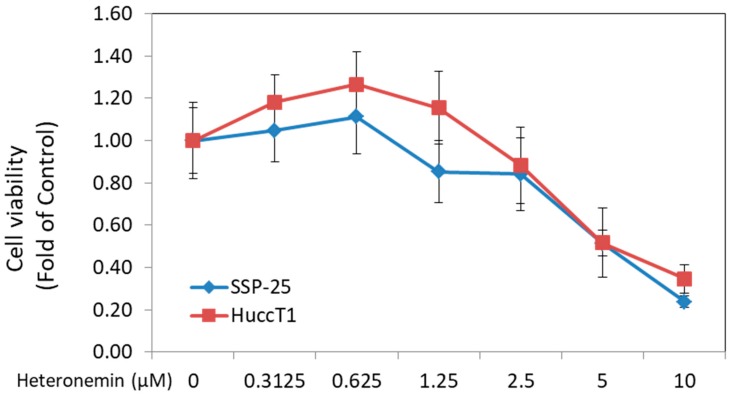
Heteronemin inhibited the cell proliferation and metastasis of cholangiocarcinoma cells in vitro. Two types of cholangiocarcinoma cells, HuccT1 cells and SSP-25 cells (2 × 10^4^ cells/well), were seeded in 96-well plates. Cells were either left untreated, or treated with different concentrations of heteronemin for 72 h with re-flashed medium containing heteronemin daily. Cell proliferation was detected by MTS Cell Proliferation Assay. Heteronemin caused a significant cytotoxic effect on both cholangiocarcinoma cell lines with IC_50_ = 4.4 μM in HuccT1 cell lines and IC_50_ = 3.9 μM in SSP-25 cells.

Heteronemin has been known to exhibit anticancer activity against several types of cancers. In this study, two cholangiocarcinoma cell lines—HuccT1 cells and SSP-25 cells—were used. Cell proliferation was detected by MTS Cell Proliferation Assay. Heteronemin caused a significant cytotoxic effect in both cholangiocarcinoma cell lines, with IC_50_ = 4.4 μM in HuccT1 cells and IC_50_ = 3.9 μM in SSP-25 cells (Figure 1).

### 2.2. Heteronemin Affects Cell Migration and Cell Adhesion in Cholangiocarcinoma Cell Lines

**Figure 2:**
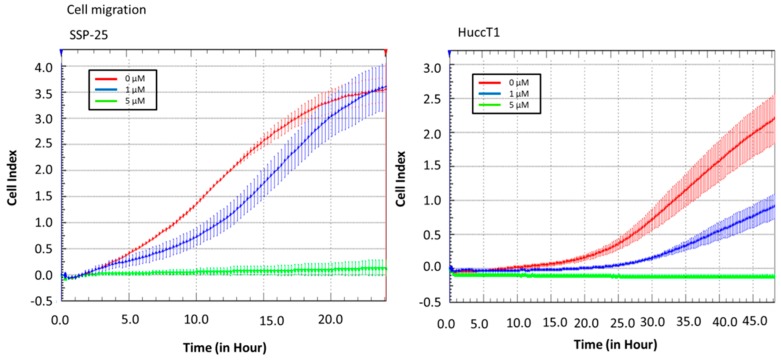
Heteronemin inhibits cholangiocarcinoma migration. Cells were added into the upper well of a real-time cell analysis (RTCA) CIM-plate to detect cell migration from the upper side to the lower side. The results show that 5 µM heteronemin reduced migration ability in cholangiocarcinoma cell lines.

**Figure 3:**
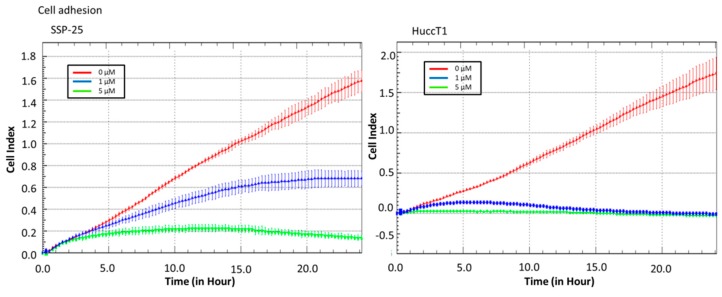
Heteronemin inhibits cholangiocarcinoma adhesion. Cells were added to an real-time-cell-analysis (RTCA) E-plate with or without heteronemin to detect the cell adhesion ability. The results show that 5 µM heteronemin reduced adhesion ability in cholangiocarcinoma cell lines.

Real-time cell analysis (RTCA) of a migration assay and an adhesion assay were performed on an xCELLigence DP device (Roche Diagnostics, Mannheim, Germany). The results show that heteronemin (5 µM) altered cell migration in both cholangiocarcinoma cell lines (Figure 2). It also inhibited cell adhesion ability (Figure 3). These results suggest that heteronemin is able to inhibit cell proliferation and metastasis in cholangiocarcinoma cells.

### 2.3. Heteronemin Regulates Expression of Genes in Cholangiocarcinoma Cell Lines

We further investigated mechanisms involved in heteronemin-induced anticancer ability in cholangiocarcinoma cells. Cells were treated with heteronemin for 24 h and total RNA was extracted. NanoString® analysis was used to detect mRNAs. A total of 770 mRNA expressions were detected by a Nanospring® nCounter PanCancer Progression Panel (NanoString Technologies, Inc., Seattle, WA, USA). The expression of 105 genes with significant change was detected in both cell lines (Table 1). The changed expression of genes, along with the biological functions that each regulates, is listed including cell growth, cell cycle, cell migration, cell invasion, epithelial-mesenchymal transition (EMT), ECM, angiogenesis, and metabolism. Heteronemin suppressed the mRNA expression of TGF-β, SMAD, and Myc. Interestingly, heteronemin decreased the expression of p53 in both cholangiocarcinoma cell lines (Table 1).

**Figure 4:**
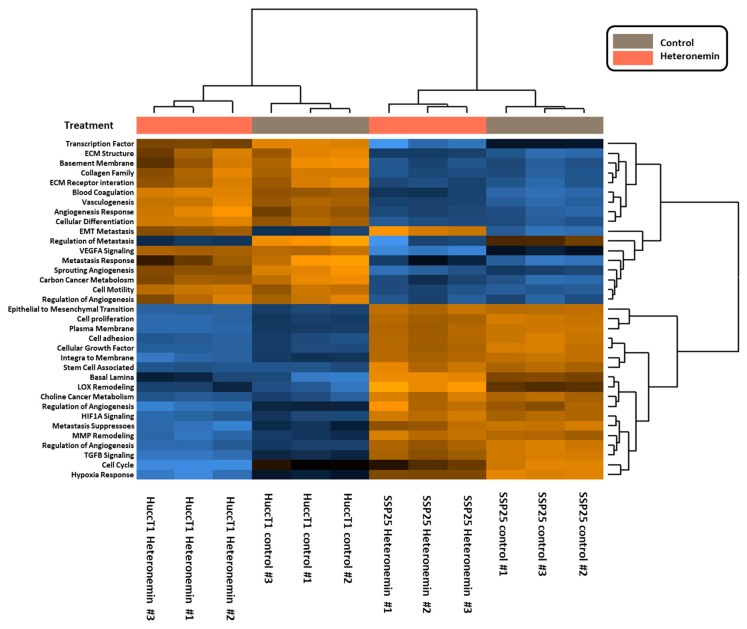
Heatmap of heteronemin regulated pathway scores. Two types of cholangiocarcinoma cells, SSP25 cells and HuccT1 cells, were treated with 5 µM heteronemin for 24 h. Total RNA was collected, and mRNAs were detected by NanoString® analysis using an nCounter PanCancer Progression Panel (NanoString Technologies, Inc., Seattle, WA, USA). The pathway scores were analyzed by nSolverTM software (NanoString Technologies, Seattle, WA, USA).

Signaling pathways induced by heteronemin were further analyzed, with pathway scores analyzed by nSolverTM software (NanoString Technologies, Seattle, WA, USA). Both cancer cell lines responded similarly to heteronemin treatment (Figure 4).

**Figure 5:**
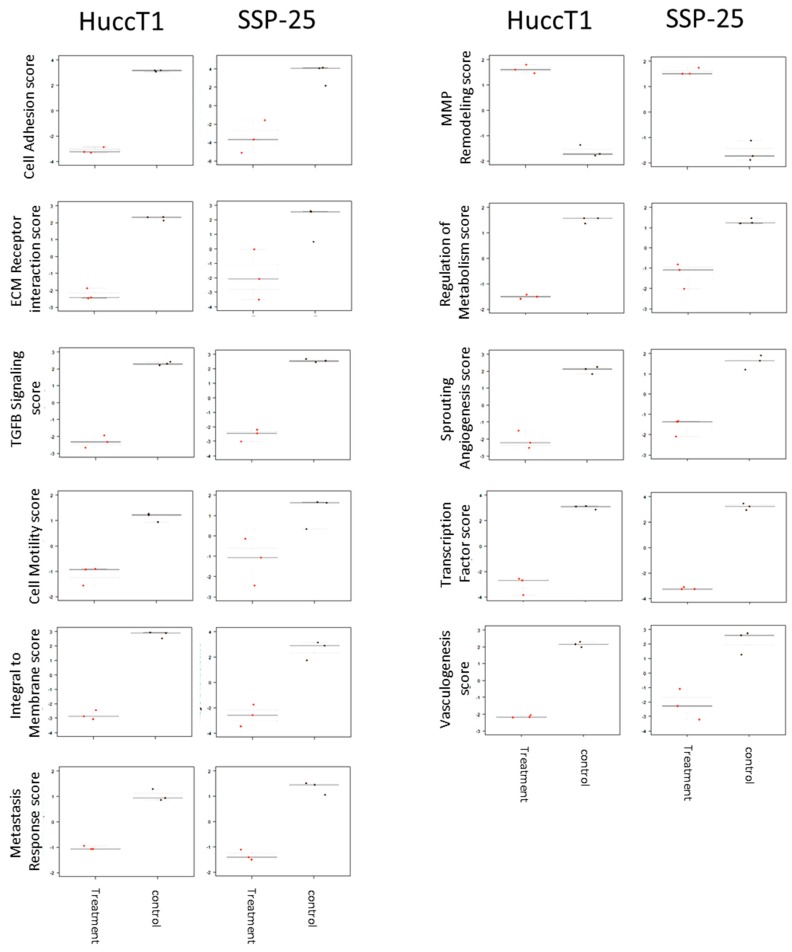
Heteronemin regulated significant pathway change in SSP25 and HuccT1 cells. SSP25 cells and HuccT1 cells were treated with 5 µM heteronemin for 24 h. Total RNA was extracted and mRNAs were detected by NanoString® analysis using an nCounter PanCancer Progression Panel. The pathway scores were analyzed by nSolverTM software.

Signal transduction pathways involved in activities of heteronemin in cholangiocarcinoma were analyzed as well. Pathway scores were analyzed by nSolverTM software (NanoString Technologies, Seattle, WA, USA). Further data analysis indicated that the treatment of cholangiocarcinoma with heteronemin altered mRNA levels of genes involved in signal transduction pathways, cell adhesion, the expression of ECM receptors, the TGF-β pathway, cell motility, membrane integration, metastasis response, MMP remodeling, the regulation of metabolism, sprouting angiogenesis, transcription factors, and vasculogenesis (Figure 5).

**Figure 6:**
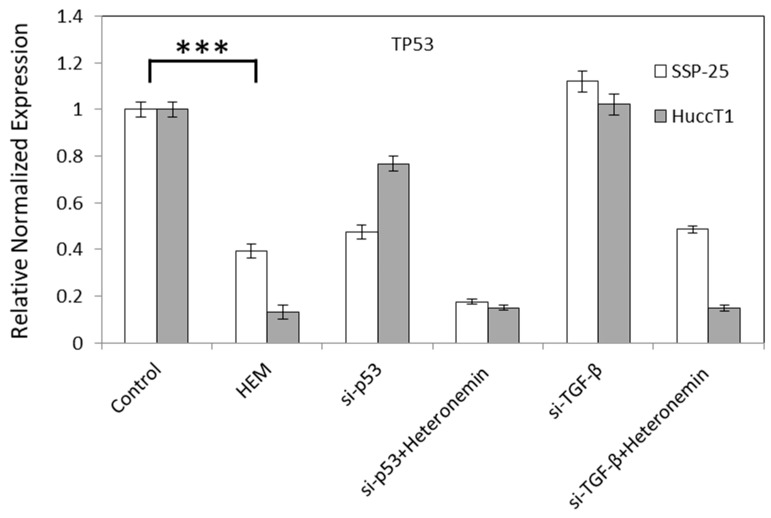
Transforming growth factor beta (TGF-β) but not p53 is involved in heteronemin-induced anti-proliferation in cholangiocarcinoma cells. SSP25 cells and HuccT1 cells were transfected with small interfering RNA (siRNA) of p53 and treated with 5 μM heteronemin for 24 h. Total RNA was extracted and qPCR was conducted for p53 and TGF-β. *p* < 0.001 (***) as compared to untreated control.

To further confirm if the inhibition of TGF-β signal without the activation of p53 plays a key role in heteronemin-induced anti-proliferation in cholangiocarcinoma, knockdown studies of TGF-β and p53 were conducted. The results shown in Figure 6 indicate that knockdown of p53 further reduced the expression of p53, but did not affect TGF-β expression in heteronemin-treated cholangiocarcinoma cells. These results suggest that heteronemin modulated multiple TGF-β-dependent signal transduction pathways to inhibit proliferation and migration in cholangiocarcinoma cells, which may not be related to p53 activation.

## 3. Discussion

The results presented in this study indicate that heteronemin inhibits proliferation and migration in two cholangiocarcinoma cell lines (Figure 1, Figure 2 and Figure 3). Heteronemin has been shown to potently suppress the viability and anchorage-independent growth of human prostate cancer cells [19]. In addition, it also activates apoptosis by the activation of both intrinsic (caspase-9) and extrinsic (caspase-8) apoptotic pathways in prostate cancer cells [19]. Heteronemin directly modulates phosphorylated talin expression through ROS generation, resulting in cell apoptosis, but it does not disturb talin/focal adhesion kinase (FAK) complex formation. Furthermore, heteronemin interferes with actin microfilament and causes morphology changes [16]. The cytotoxic effect of heteronemin is associated with the oxidative stress and induction of phosphorylated talin expression [16]. In addition, heteronemin inhibits the phosphorylation of the c-Met/src/STAT3 signaling axis and the expression of signal transducer and activator of transcription (STAT)3-regulated genes including Bcl-xL, Bcl-2, and Cyclin D1. It efficiently antagonizes the hepatocyte growth factor (HGF)-stimulated c-Met/STAT3 activation, as well as proliferation and colony formation in refractory prostate cancer cells [19]. Our results indicate that heteronemin concurrently inhibits TGF-β expression with anti-proliferation, anti-migration, and adhesion (Figure 2 and Figure 3 and Table 1).

Knockdown of TGF-β further enhanced heteronemin-induced anti-proliferation (results not shown). Our [4] and others’ results [5,7,8,20,21] indicate that TGF-β acts as a tumor promoter in the progressive stages of cancers to support tumor cell motility, survival, invasion, metastasis, and immune evasion. The connection between the signaling of p53 and TGF-β has been well-demonstrated [22]. TGF-β is able to induce NADPH oxidase 4 (Nox4)-dependent, p21 (Cip1)-dependent, p15 (Ink4b)-dependent, and ROS-dependent—but not p53- and p16 (Ink4a)-dependent—senescence arrest in well-differentiated hepatocellular carcinoma (HCC) cells [9]. In addition, the antitumor effect of TGF-β signaling stabilizes wild-type p53 accumulation and recruitment to its DNA-binding sites on chromatin. The inactivation of p53 interrupts TGF-β-induced cellular activities. However, crosstalk between p53 and TGF-β signaling demonstrates that p53 can act as a component of SMAD complexes to participate in the stabilization of SMAD-DNA complexes and modulate various tumor suppressor genes [23,24,25,26,27]. Knockdown p53 did not reduce or affect the abundance of TGF-β (Figure 6), indicating that p53 may not associate with TGF-β to form a complex and stabilize p53. In addition, heteronemin reduced p53 expression (Table 1). Together, the results suggest that p53 is not involved in the heteronemin-induced anti-proliferation in cholangiocarcinoma examined in this study.

In advanced stages of cancers, TGF-β also functions as a tumor promoter to support the proliferation, metastasis, and immune evasion of cancer cells [5,6,7,8]. Initiating in the binding with receptor TGF-βRII, TGF-β forms a heterotetrameric complex with TGF-βR1. Subsequently, TGF-βRII phosphorylates TGF-βR1, then recruits and phosphorylates cytosolic transcription factors SMAD2 and SMAD3 [10,11,12]. Phosphorylated SMAD2/3 form a complex with co-SMAD (SMAD4) after dissociation from the TGF-β receptors. The formed transcriptional complex translocates into the nucleus and regulates the expression of numerous target genes [13,14]. Furthermore, TGF-β signaling also involves several non-SMAD pathways, such as phosphoinositide 3-kinase (PI3K)/AKT, INK/p38, Ras-ERK, and RhoA pathways [5,28]. The cooperation between SMAD and non-SMAD pathways determines the ultimate consequence of cellular reactions to TGF-β.

In conclusion, our findings suggest that heteronemin may activate a novel signaling pathway to inhibit the expression of TGF-β, SMAD, and their signaling pathway in order to suppress the proliferation, invasion, and adhesion of cholangiocarcinoma. Apparently, p53 does not have a significant role in heteronemin-induced activities.

## 4. Materials and Methods

### 4.1. Cell Culture

Two human cholangiocarcinoma cell lines (SSP-25 and HuccT1) were purchased from RIKEN Bioresource Center (Ibaraki, Japan) and maintained routinely in RPMI-1640 or a minimum essential medium (MEM) containing 10% fetal bovine serum (FBS) and 1% P/S solution (Invitrogen) in a 5% CO2 incubator at 37 °C. Cholangiocarcinoma cells were incubated in a 6-well tissue culture dish for 24 h. Negative control (NC) small interfering RNA (siRNA), specific p53 (sc-29435, California, USA), and TGF-β (sc-270322, California, USA) siRNA transfection reagent complexes were mixed with PolyJet™ reagent (SignaGen Laboratories, Rockville, MD, USA) according to the manufacturer’s recommendation, and were added to the cells.

### 4.2. MTS Cell Proliferation Assay

Cells (2 × 10^4^ cells/well) were seeded in 96-well plates and treated with or without heteronemin (Sigma, St. Louis, MO) for 72 h. Cell proliferation was determined by incubating cells with 200 mL of fresh medium containing MTS (ab197010, Abcam, Cambridge, MA 02139, United States) for 4 h at 37 °C. The plates were read using a microplate reader (VersaMax™ Tunable Microplate Reader, Molecular Devices, Sunnyvale, CA) to measure the absorbance at 490 nm. Triplicate wells were assayed for each experiment, and three independent experiments were performed. The values of IC_50_ were evaluated using the method by Chou and Talalay with CompuSyn freeware (ComboSyn Inc., Paramus, NJ. 07652 USA).

### 4.3. Cell Migration and Cell Adhesion Assay

Real-time cell analysis (RTCA) of a migration assay and an adhesion assay was performed on the xCELLigence DP device (Roche Diagnostics, Mannheim, Germany), as described in the supplier’s instruction manual. The migration assay used a CIM-plate, and the adhesion assay used an E-plate. In the migration assay, cells treated with or without heteronemin in serum-free RPMI-1640 medium were added to the upper chamber of a two-chamber device. They were separated by a porous membrane and RPMI-1640 medium with 10% FBS at the down-slide as a factor affecting chemotaxis cell migration. When cells migrated from the up-side to the down-side, the detector at the down-side would detect signals, and indices were measured every 15 minutes for up to 48 h using RTCA software (version 1.2, Roche Diagnostics, Risch-Rotkreuz, Switzerland). In the adhesion assay, cells treated with or without heteronemin in RPMI-1640 medium with 10% FBS were added to the well of an E-plate, and indices were measured every 15 minutes for up to 24 h with RTCA software. Triplicate wells were assayed for each experiment, and three independent experiments were performed.

### 4.4. RNA Isolation and NanoString® Analysis

Cells (2 × 10^6^ cells/well) seeded in a 6-well plate overnight were treated with 5 µM heteronemin for 24 h. Total RNA was extracted using RNeasy Micro Kit (Qiagen, Venlo, The Netherlands). For quality assurance, RNA samples with an RNA Integrity Number (RIN) greater than 5 were used for NanoString® analysis. An nCounter PanCancer Progression Panel was used. Triplicate wells were assayed for each experiment, and three independent experiments were performed.

### 4.5. Quantitative Real-Time PCR

Total RNA was extracted and genomic DNA was eliminated with a Illustra RNAspin Mini RNA Isolation Kit (GE Healthcare Life Sciences, Buckinghamshire, UK). One microgram of DNase I-treated total RNA was reverse-transcribed with a RevertAid H Minus First Strand cDNA Synthesis Kit (Life Technologies Corporation, Carlsbad, CA, USA) into cDNA. It was then used as the template for real-time PCR reactions and analysis. The real-time PCR reactions were conducted using a QuantiNovaTM SYBR® Green PCR Kit (QIAGEN, Valencia, CA, USA) on a CFX Connect™ Real-Time PCR Detection System (Bio-Rad Laboratories, Inc., Hercules, CA, USA). This involved an initial denaturation at 95 °C for 5 min, followed by 40 cycles of denaturing at 95 °C for 5 s, and combined annealing/extension at 60 °C for 10 s, as described in the manufacturer’s instructions. The primer sequences were as follows: 18S rRNA forward 5′-GTAACCCGTTGAACCCCATT-3′ and reverse 5′-CCATCCAATCGGTAGTAGCG-3′; TP53 forward 5′-AAGTCTAGAGCCACCGTCCA-3′ and reverse 5′-CAGTCTGGCTGCCAATCCA-3′; TGF-β forward 5′-TACAGACCCTACTTCAG-3′ and reverse 5′-AAATCTTGCTTCTAGTT-3′. The relative gene expression, normalized to the internal control 18S rRNA, was calculated based on the ΔΔCT method, and the fidelity of the PCR reactions was determined by melting temperature analysis.

### 4.6. Quantification of Results and Statistical Analysis

Densities of gene expression of quantitative real-time PCR were analyzed by IBM SPSS Statistics software version 19.0 (SPSS Inc., Chicago, IL, USA). Student’s *t*-test determined significance, with *p* value < 0.001.

## Figures and Tables

**Table 1:** Significant expression changes of genes affected by heteronemin in cholangiocarcinoma cell lines.

Gene Name	HuccT1: Treatment vs. Control	SSP-25: Treatment vs. Control	Gene Name	HuccT1: Treatment vs. Control	SSP-25: Treatment vs. Control	Gene Name	HuccT1: Treatment vs. Control	SSP-25: Treatment vs. Control
*HSPB1*	88.233	7.551	*CD24*	−1.568	−3.733	*IL1B*	−2.096	−2.691
*HMOX1*	71.408	3.531	*NRP1*	−1.572	−2.019	*FSTL1*	−2.097	−1.956
*SNAI1*	34.199	4.227	*PTTG1*	−1.572	−3.149	*MGAT5*	−2.121	−1.854
*SERPINH1*	25.425	2.579	*RAC2*	−1.587	−2.025	*CHD4*	−2.128	−2.105
*CREBBP*	13.219	2.045	*HPSE*	−1.593	−2.863	*SMC3*	−2.135	−2.116
*COL7A1*	9.126	2.896	*RORB*	−1.597	−3.446	*EPHA1*	−2.15	−2.39
*JUN*	8.583	4.375	*ITGB6*	−1.602	−3.286	*CEACAM1*	−2.152	−1.723
*CLDN4*	6.036	1.97	*EPHB4*	−1.626	−1.674	*HOXB3*	−2.182	−2.682
*NDRG1*	4.916	2.429	*GPR124*	−1.633	−1.831	*VAV3*	−2.187	−2.392
*EIF2AK3*	4.619	3.731	*LAMC1*	−1.637	−1.767	*ILK*	−2.22	−2.484
*IL11*	4.556	1.874	*MYLK*	−1.641	−4.584	*C3*	−2.278	−1.906
*BTG1*	4.158	2.085	*TMEM30B*	−1.641	−2.574	*SLC2A1*	−2.298	−2.845
*NOTCH1*	4.065	3.544	*CLIC4*	−1.667	−2.164	*BMP5*	−2.302	−7.016
*FGFR1*	4.061	1.834	*P3H2*	−1.67	−2.335	*DICER1*	−2.32	−1.83
*VEGFA*	3.731	2.412	*SPOCK3*	−1.675	−1.897	*EPHA2*	−2.444	−2.341
*COL6A2*	3.429	2.169	*EIF4E2*	−1.678	−1.966	*CAV1*	−2.448	−7.07
*CAMK2D*	3.256	3.202	*ENO1*	−1.685	−2.548	*LDHA*	−2.458	−5.537
*HKDC1*	3.224	1.896	*FREM2*	−1.685	−2.227	*KDM1A*	−2.498	−2.083
*SIRT1*	3.183	1.756	*CCDC80*	−1.7	−4.522	*IGFBP4*	−2.533	−2.556
*NFAT5*	3.113	3.759	*CHI3L1*	−1.708	−2.561	*GALNT7*	−2.553	−2.494
*DST*	2.832	3.179	*PXDN*	−1.708	−3.924	*AHNAK*	−2.639	−2.638
*LAMA5*	2.813	1.904	*CDH2*	−1.711	−1.555	*LRG1*	−2.66	−3.45
*CD44*	2.7	1.933	*RBL1*	−1.713	−3.098	*VWA2*	−2.677	−3.884
*LTBP4*	2.523	2.62	*ICAM1*	−1.717	−2.351	*NFKB1*	−2.724	−2.311
*HSP90B1*	2.346	1.771	*ARHGDIB*	−1.719	−3.341	*GTF2I*	−2.762	−1.824
*SETD2*	2.341	3.568	*RBM47*	−1.728	−2.989	*TP53*	−2.782	−5.449
*ZEB2*	2.292	1.755	*CALD1*	−1.732	−1.593	*DAG1*	−2.824	−3.982
*ADAM17*	2.271	2.214	*NME1*	−1.74	−2.934	*CMA1*	−2.854	−3.277
*CDKN1A*	2.215	2.449	*MMP17*	−1.746	−2.125	*ITGA6*	−2.938	−2.455
*ADM2*	2.118	1.886	*RB1*	−1.757	−1.919	*TFDP1*	−2.957	−3.597
*PLEKHO1*	2.087	3.115	*SDC4*	−1.763	−1.732	*ITGB8*	−2.972	−3.313
*MYC*	2.08	2.247	*RUNX1T1*	−1.783	−5.442	*PDGFC*	−3.001	−3.104
*HIPK1*	2.04	1.615	*CGN*	−1.791	−2.974	*KCNJ8*	−3.103	−6.262
*PLXND1*	1.813	1.683	*PKM*	−1.805	−2.413	*CDH11*	−3.256	−3.584
*SERINC5*	1.796	2.272	*TACSTD2*	−1.831	−3.024	*EDN1*	−3.259	−2.311
*ADAMTS1*	1.79	6.932	*SACS*	−1.832	2.214	*PLS1*	−3.263	−2.762
*PLAUR*	1.778	2.422	*ITGA3*	−1.845	−1.869	*F3*	−3.458	−14.714
*SRPK2*	1.656	2.519	*HK2*	−1.884	−1.628	*SMAD3*	−3.521	−4.024
*MMP1*	1.612	5.34	*ALOX5*	−1.887	−3.82	*THBS1*	−4.102	−8.476
*ACHE*	1.59	2.054	*RBL2*	−1.915	−2.354	*TGFBR2*	−4.281	−1.759
*PFKFB4*	1.588	1.725	*GLYR1*	−1.932	−1.868	*PTX3*	−4.289	−1.998
*ENPEP*	−1.516	−4.42	*STAT3*	−1.945	−1.702	*BMP4*	−4.641	−3.477
*IL1A*	−1.516	−1.985	*SRF*	−2.057	−2.931	*VCAN*	−5.209	−3.999
*ITGB2*	−1.521	−3.986	*IL13RA2*	−2.058	−2.512	*TGFB2*	−5.482	−4.253
*SNRPF*	−1.526	−1.828	*DEN*	−2.059	−1.524	*LAMA3*	−5.659	−2.226
*NRP2*	−1.527	−1.952	*COL6A1*	−2.079	−1.568			
*CDC42*	−1.545	−1.584	*CDS1*	−2.092	−2.943			

HuccT1 cells and SSP25 cells were treated with 5µM heteronemin for 24 h. Total RNA was collected, and mRNAs were detected by NanoString® analysis. Expression change greater than 1.5 fold is considered significant.
